# Determinants of Fast-Food Consumption in Romania: An Application of the Theory of Planned Behavior

**DOI:** 10.3390/foods10081877

**Published:** 2021-08-13

**Authors:** Abigaela Bîlbîie, Elena Druică, Remus Dumitrescu, Daniela Aducovschi, Robert Sakizlian, Monica Sakizlian

**Affiliations:** 1 The Academy of Economic Studies, Faculty of Theoretical and Applied Economics, 010552 Bucharest, Romania; bilbiieabigaela@gmail.com; 2 Centre for Applied Behavioral Economics, Department of Economic and Administrative Sciences, University of Bucharest, 030018 Bucharest, Romania; 3 Department of Physical Education, University of Bucharest, 030018 Bucharest, Romania; remus.dumitrescu@unibuc.ro (R.D.); daniela.aducovschi@unibuc.ro (D.A.); robert.sakizlian@unibuc.ro (R.S.); monica.sakizlian@unibuc.ro (M.S.)

**Keywords:** fast-food consumption, behavioral determinants of fast-food consumption, theory of planned behavior, young adults and fast-food consumption

## Abstract

This study explores drivers of fast-food consumption in Romania using the Theory of Planned Behavior. We analyze 532 responses to an online survey and use partial least squares path modeling to estimate the relationships between the intention to consume fast food and its possible determinants. Our results show that the most significant predictor is the subjective norms (injunctive norms: β = 0.218, *p* < 0.001; descriptive norms: β = 0.192, *p* < 0.001). Among the affective and cognitive attitudes, only the latter is statistically significant in predicting the intention (β = 0.088, *p* = 0.020), while perceived behavioral control is not significantly associated with intention toward fast-food consumption. We explain how our results can help policymakers to design better interventions on public health concerns about fast-food consumption and population obesity, especially children obesity.

## 1. Introduction

While children tend to consider eating at a fast-food restaurant a special event [1], obesity and other food-related diseases are on the rise [2,3]. In 2016, more than 340 million children and young adults between 5 and 19 years old were overweight or obese, and in 2020, a number of 39 million children under the age of 5 were diagnosed as obese or overweight [4]. Since 1975, global obesity has nearly tripled, and approximately 1.9 billion adults over the age of 18 are overweight, and over 650 million of them are obese [4]. According to the authors of [5], abdominal obesity was associated with fast-food consumption. Sandwich intake was linked to obesity and overweight in 35% of the cases, fried chicken in 40% of cases, and pizza in more than 80% of cases. With low nutritional properties [6], fast food includes burgers, hot chips/French fries, fried chicken, certain mass-produced pizzas [7], and also doughnuts [1], being currently served by 897.683 restaurants worldwide with a market size of USD 860 billion and more than 14 million [8].

There is significant scientific research on fast-food consumption among adolescents, driven by the high tendency of “people in their teens and twenties” to consume energy-dense food products [1]. The preferences for this type of food are more prevalent in low- and middle-income countries where they signal a “nutritional transition” from the country’s traditional food to a Westernized diet [9], which includes excessive consumption of high-fat dietary products, processed meats, sweets and soft drinks [10]. These energy-dense foods are low in fiber and essential nutrients, with a high amount of salt, sugar, and refined carbohydrates [1,11], resulting in population overweight, child obesity, headache, depression, dental distress, acne, heart disease or stroke, high blood pressure, high cholesterol, blood sugar spike, bloating and puffiness, and insulin resistance [12,13].

The “nutritional transition” among young adults exposed to a Westernized culture impacts Romania as well. Romania is a middle-income country, where 1.2 million people accounting for 6% of the entire population, report themself as fast-food consumers. Among them, 200.000 people eat fast food daily [14]. Between 2017 and 2019, the rate of fast-food consumption among Romanians has doubled, reaching a total market size of 14 billion RON (an equivalent of almost 3 billion euro). The most favorite fast-food products bought by Romanians are pizzas, burgers, French fries, sandwiches, and shawarmas [15].

Similar to other countries, fast-food consumption in Romania leads to health concerns such as diabetes, obesity, and cardiovascular diseases. Even if the Romanian obesity rate is the lowest among other European countries, the children’s obesity rate has increased by 15% in the last decade [16]. In 2016, Romania was included in the top five countries with the highest risk of cardiovascular diseases, with a mortality of 58% for both sexes [17]. The prevalence of diabetes in adults is 8.8% [18], with prevalence in children aged 0 to 14 years old 5.4 per 100.000 inhabitants. While there is no significant difference in the prevalence of diabetes between males and females, in the case of overweight and obesity, males are more prone to be overweight (65.2%) than females (56.5%) but do slightly better than females at obesity rates (21.8% compared to 24.9% for females [19]). Poor nutrition, insufficient consumption of vegetables and fruits, and excessive consumption of sugar and salt are one of the main causes responsible for almost 30% of all fatalities in Romania. The figures point toward the need for informed interventions aimed to reduce fast-food consumption as one important source of the above-discussed morbidity.

To address this concern, our paper aims to identify Romanians’ drivers to buy and consume fast food, using the theory of planned behavior (TPB) as theoretical background and a sample of 532 respondents to an online survey. Based on partial least squares path modeling conducted in WarpPLS, we not only assess the statistical relevance of the predictors proposed by TPB but also identify the most suitable determinant for practical interventions aimed to reduce intention to consume fast food.

The rest of the paper is organized as follows: Section 2 provides information about the relevance of the TPB in predicting food choice and sets the research hypotheses. Section 3 discusses the materials and the method, while Section 4 presents the results. The discussions section presents theoretical as well as practical implications of the findings along with the limitations of the study and future directions of research. Section 6 briefly concludes.

## 2. Literature Review

### 2.1. Theoretical Framework

The theory of planned behavior framework assumes that attitudes, subjective norms, and perceived behavioral control predict behavioral intention, which further determines the corresponding actual behavior [20]. The popularity of this theory is explained by its ability to explain a wide range of human behaviors, using this compact structure of attitudes, subjective norms, and individual control.

*Attitude* refers to a favorable or an unfavorable assessment of a certain outcome [21] related to the performance of a certain behavior (for example, eating fast food), and it can be divided into two categories [7,22]: *affective attitude* (the emotional component reflecting an individual’s feelings about something) and *cognitive attitude* (the rational component reflecting an individual’s knowledge or beliefs about something). Favorable attitudes support behavioral intention, while negative attitudes hinder it.

Two basic categories of *subjective norms (SN)* are discussed in the work of [23]: injunctive and descriptive. *Injunctive norms* refer to an individual’s perception that society or a specific reference group will approve or disapprove their behavior, and *descriptive norms* highlight the social pressure an individual may experience comparing their behavior in a certain situation with what is generally recognized as normal conduct. In different words, injunctive norms refer to what we think that other people expect us to do, while descriptive norms refer to the perception of what other people actually do, this setting the norm with respect to a certain behavior [24]. This partition is also supported by the authors of [25], where subjective norms are split into a category that involves information (injunctive) and another category that involves peer pressure (descriptive). According to the TPB, subjective norms of both types are expected to support behavioral intention if they signal that the behavior is desirable. However, previous research shows that the mechanisms that underline these influence are different: injunctive norms acts through all five types of social influence discussed by the authors of [26], while descriptive norms drive the behavior through only two of them [23].

*Perceived behavioral control* captures people’s self-reported control on performing a given behavior [27] through its two dimensions: *perception of control*, which focuses on factors from the outside of the individual (external control), such as others’ behavior, accessibility, task difficulty, etc., and *self-efficacy*, which involves factors that come from the inside of the individual (internal control), such as motivation, ability, or personality [28]. The TPB framework assumes that both dimensions of the perceived behavioral control support behavioral intention in the case of positive behaviors and hinder the intention of undesirable behaviors.

### 2.2. Hypotheses Development

TPB was successfully applied to explain a large variety of behavioral intentions and actual behaviors, from recycling, travel, technology adoption, protection of privacy to smoking, drinking, the use of health services, breastfeeding, and drug use [27,29]. Healthy eating behaviors [20], genetically modified food consumption [30], organic food consumption [31], consumption and purchasing of halal food [32,33], sustainable food consumption [34,35] food neophobia and ethnic food consumption [36], functional food purchasing [37], and fast-food consumption [7,11,38,39] are food-related applications of the TPB.

According to the authors of [20], subjective norms and perceived behavioral control predict 30% of the variance in intention to adopt healthier eating behavior. In the application of TPB on genetically modified food consumption, the model had an explanatory power of 44.4%, with attitude being the strongest predictor [30], while in the study conducted on organic food consumption [31], subjective norms were the most significant predictor of the intention to consume. In the case of functional food consumption [37], both attitude and subjective norm had the strongest influence on intention. As regards the consumption and purchasing of halal food, [32] indicated that TPB was able to explain 29.1% of the variance in intention, and all the TPB’s constructs were significant predictors of halal food consumption, while [33] argued that the only attitude has the highest influence on halal food consumption and the model has lower explanatory power, of only 24%. Other studies also found attitude as the strongest predictor [40] and positively related to behavioral intention [7]. All of the TPB’s constructs have the power to explain on average 48% of intention to buy and consume sustainable food, according to the work of [34], although [35] suggest that social norms have the strongest ability to predict sustainable food consumption. As for the study conducted on food neophobia and ethnic food consumption [36], the TPB model was able to explain 42.1% of the variance in the intention to consume Dayak food, attitude having the strongest effect on intention compared to subjective norms and perceived behavioral control.

In an attempt to better understand how attitude influences individual behavior, previous studies [7,41,42] have debated the existence of two dimensions of the construct: affective and cognitive. The main findings of these studies indicated that measures of cognitive and affective attitudes load on distinct factors and are differently associated with intention. In the case of fast-food consumption, although mean scores for both cognitive and affective attitudes show a slightly negative attitude toward fast-food consumption [7], the findings revealed by the same study suggest that only cognitive attitude was notably significant in the prediction of the intention to consume fast-food. The context for the failure of affective attitude to predict fast-food consumption is explained by the authors of [43,44], who highlight the fact that an affective attitude is usually a predictor of healthy food consumption rather than a snack or sweet food consumption. In line with these considerations, the following research hypotheses are assumed to hold in the Romanian case too:

**Hypothesis** **1a** **(H1a):** *Affective attitudes are positively related to behavioral intention to consume fast food.*

**Hypothesis** **1b** **(H1b):** *Cognitive attitudes are positively related to behavioral intention to consume fast food.*

**Hypothesis** **1c** **(H1c):** *Cognitive attitudes are stronger predictors of the behavioral intention to consume fast food than affective attitudes.*

Subjective norms, especially those influenced by reference groups, such as friends, have been found to strongly affect the intention to consume fast food in Iran [45]. Both injunctive and descriptive norms were found as strong determinants of fast-food consumption in The Nederlands, although the effect sizes were small [46]. Another study conducted in Australia found that injunctive norms are predictors of intention to consume fast food [38], while other studies employing the TPB to examine dietary behaviors reveal that subjective norms are a stronger predictor of the intention in adolescents samples compared with adults samples situations [47]. Although the assumptions of the theoretical model, as well as previous research, suggest that both injunctive and descriptive norms positively support the behavioral intention to consume fast food, we expect that the two components of the subjective norms have different contributions. As the authors of [26] state, what others do and what others approve are different sources of human motivation. While descriptive norms refer to what is commonly done and indicate what is effective and adaptive, injunctive norms are moral drivers and promise social rewards. There is conflicting evidence regarding the contribution of each type of subjective norm in predicting the intention to perform a certain behavior. While some studies show that injunctive norms account for a larger variability in the behavioral intention and behavior than descriptive norms do [48,49], other contributions show the opposite [50]. As a consequence, we will assume that the descriptive and the injunctive norms are equally strong in predicting the behavioral intention to consume fast food and set the following research hypotheses:

**Hypothesis** **2a** **(H2a):** Injunctive norms are positively related to behavioral intention to consume fast food.

**Hypothesis** **2b** **(H2b):** *Descriptive norms are positively related to behavioral intention to consume fast food.*

**Hypothesis** **2c** **(H2c):** *The injunctive and the descriptive norms are equally strong predictors of the behavioral intention to consume fast food.*

Reference [23] stated that a person’s intention to act in a certain way is influenced by the perceived behavioral control, through both external control, represented by perceptions of control, and internal control, represented by self-efficacy. As for the influence exerted, perceived behavioral control has between moderate and strong influence on behavioral intention [47]. Furthermore, [1] shows that behavioral intention is strongly associated with perceived behavioral control, a result confirmed by the authors of [51] that shows that higher levels of perceived behavioral control are related to lower levels of intention to consume fast food. In terms of dimensions of the perceived behavioral control, the TPB framework assumes that both perceptions of control and self-efficacy are negatively associated with the intention to perform undesirable behaviors, as is the case with fast-food consumption. Previous findings identified these constructs as having, among all TPB determinants, the strongest influence on people’s intention for healthy eating [52]. Both of them positively relate to the intention to reduce fast-food consumption, with the perception of control as the strongest predictor [53]. Other studies emphasize the importance of self-efficacy in improving adherence to healthy diets [54], which in turn suggests a negative association with the intention to consume unhealthy food. We, therefore, set the following hypotheses:

**Hypothesis** **3a** **(H3a):** *Perception of control is negatively related to the intention to consume fast food.*

**Hypothesis** **3b** **(H3b):** *Self-efficacy is negatively related to the intention to consume fast food.*

**Hypothesis** **3c** **(H3c):** *Perception of control is a stronger predictor of the intention to consume fast food than self-efficacy.*

Self-identification has been found as the strongest predictor of behavioral intention than other TPB constructs, especially in the domain of food choice and consumption [55]. The result is also supported by the authors of [7], suggesting that people who self-identify as healthy eaters have weaker intentions to consume fast food frequently.

**Hypothesis** **4** **(H4):** *Self-identification as a healthy eater is negatively related to the behavioral intention to consume fast food.*

## 3. Materials and Methods

### 3.1. Data

Data were collected between November 2020 and February 2021, using a self-reported questionnaire made on Google Forms and sent to individuals through different social platforms, such as Facebook, WhatsApp, and e-mail, via convenience and snowball sampling. The Ethical Committee of the University of Bucharest approved the research (decision no 146/07.07.2021). Before completing the questionnaire, the respondents were informed that their participation in this study is anonymous and entirely voluntary, and by filling in the survey, they provide consent to participation in the study. The minimum sample for a significance level of 0.05 and a power level of 0.990 was 410 if calculated using the inverse square root method and 392 if calculated using the gamma-exponential method.

### 3.2. Measurement

The aim of this study is to explore what are the determinants of fast-food consumption among Romanians and to what extent they influence behavioral intention. We use the theory of planned behavior and replicate the research of [7] on fast-food consumption in Australia, from which we translated the constructs. The reason for this approach comes from the fact that the original paper builds on a strong and previously tested theoretical background, enriched with control variables rooted in core principles of human psychology. Consequently, we expect that the same research design is appropriate regardless of the country. However, a direct comparison between Romania and Australia can bring more. Even if both countries have been going through a rapid “nutrition transition”, Romania started to adopt a Western diet pattern almost thirty years later than Australia, after the fall of the communist regime [56,57]. If we find that, despite the time gap and the different geographical locations of the two countries, the drivers of fast-food consumption in Romania match the Australian ones, the extant literature can suggest research and inform practical interventions in Romania that align with those adopted worldwide.

#### 3.2.1. Behavioral Intention to Consume Fast Food

The survey measured the intention to consume fast food, attitudes and subjective norms, and the respondents’ perceived behavioral control over fast-food consumption. Intention to consume was measured using two 7-point Likert-scale items concerning the respondents’ likelihood to buy or consume fast food over the next month. To assess the key predictors of behavioral intention, namely attitude, subjective norm, and perceived behavioral control, we divided every dimension in two, as the authors of [7] suggest, and as can be observed in Table 1.

#### 3.2.2. Attitudes

Attitudes were split into cognitive and affective. The cognitive attitudes were measured using the words *“To me, eating fast food frequently is…”* followed by a list of five sets of adjectives (e.g., harmful–beneficial, cheap–expensive, etc.), all rated on a 7-point scale. To measure affective attitudes, we used the following: *“Eating fast food frequently makes me…”* followed by twelve sets of adjectives (e.g., happy–unhappy, worried–calm, etc.) rated on a 7-point scale.

#### 3.2.3. Subjective Norms (SN)

Subjective norms were also split into injunctive norms and descriptive norms. Injunctive norms refer to the individual’s perceptions about what others expect regarding fast-food consumption (e.g., *“Those close to me expect me to eat fast food regularly”*), and were measured using 2 items presented on a 7-point scale (1—definitely false to 7—definitely true). In order to capture how respondents perceive their peer group’s behavior (descriptive norms) regarding fast-food consumption (e.g., *“Those who are close to me eat fast food regularly”)*, another 2 items rated on the same 7-point scale were added.

#### 3.2.4. Perceived Behavioral Control (PBC)

In line with [7], perceived behavioral control was measured by using two sub-dimensions: perceptions of control and self-efficacy. Perceptions of control were assessed using two items (e.g., *“I have complete control over the number of times I will eat fast food over the next month.”)* on a 7-point scale between 1 (definitely false) and 7 (definitely true). Self-efficacy was also measured using two items, both on a 7-point scale (e.g., *“It would be impossible for me not to eat fast food regularly over the next month”)*.

#### 3.2.5. Control Variables

Apart from TPBs dimensions, and following the research design adopted by the authors of [7,38], we added as controls: Consideration of Future Consequences Scale (CFC) [58,60], Fear of Negative Evaluation Scale (FNE) [59], the extent to which the respondents identify themselves as healthy eaters, facilitating factors and impeding factors. A detailed review of the theoretical background that underlines these influences is presented in the work of [7,38,61].

According to the authors of [7], facilitating factors, such as not cooking at home, eating alone, nearby fast-food restaurants, etc., influence people to consume fast food, while the impeding factors, such as concerns about weight, health, or fast-food cost, should inhibit to some extent the appetite for fast-food products.

The relevance of CFC in foregoing eating behaviors was proven in extant research [58,62], although in the concrete case of fast-food eating, the concern for the future negative effects of the dense food can be offset by the short-term benefits experienced by the eater [7,38,63]. The fear of negative evaluation, defined as the extent to which an individual fears disregard from others [59], was already identified as an inhibitor of food consumption [64,65]. Although originally accounted for as an important predictor of eating disorders [64,66], other authors took it into consideration as a control variable in other food-related contexts, including fast-food consumption [7,61]. To the CFC Scale’s original items, another two items were added for an appropriate investigation of CFC in connection with diet (i.e., “I often avoid certain foods because I am concerned about my health.” And “I usually choose food because it is convenient or tasty rather than because it is good for my health.”) We used the original FNF scale comprising 12 items (examples are *“I am unconcerned even if I know people are forming an unfavorable impression of me”* or *“I am usually worried about what kind of impression I make”*.)

Furthermore, apart from CFC Scale and FNE Scale, we used as control variables facilitating and impeding factors of fast-food consumption previously accounted for by the authors of [67,68,69]. We operated with the 7 items out of 24 found as statistically significant in previous research, 4 items to measure facilitating factors (e.g., *“I am more likely to eat fast food if I have a little spare time.”),* and 3 items to measure impeding factors (e.g., *“I feel guilty if I eat fast food.”)* [7].

Finally, using a 4-item scale, we measured how respondents self-identified as healthy eaters by asking questions such as “I think of myself as someone who is concerned with the health consequences of what I eat” and “I think of myself as a healthy eater”.

### 3.3. Method

Supported by the lack of normality of our data, we use a partial least squares algorithm [70] that provides information about how much variance in the result can be explained by the TPB predictors along with the control variables. The algorithm that underlines partial least squares estimation is iterative, composed of two parts: a measurement (or outer) model that provides scores of the latent constructs, and a structural (inner) model, that assesses the relationships among variables. To conduct our analysis, we used WarpPLS software, version 7.0.

## 4. Results

Our sample consists of 532 respondents (370 women and 162 men), with an average age of 22 years. A total of 83% of the respondents have ages between 18 and 24 years old, this being the predominant age group in our sample. A total of 67% of the respondents have their provenience in urban areas, and 89.3% of them declared they are students. This can explain why more than half of them have an income under 1400 lei (minimum net wage in Romania). A complete sample description is available in Table 2.

### 4.1. The Measurement (Outer) Model

After a preliminary investigation, the Consideration of Future Consequences Scale was excluded since only one item has a loading over 0.7 [71]. Out of all latent variables that measure the TPB dimensions, we have to drop three items from affective attitude, two items from cognitive attitude, and both items that measured self-efficacy since the items’ loading were below the 0.7 threshold. The final structure of the latent constructs is presented in Appendix A.

Table 3 presents the reliability of measurement for each remained construct. The composite reliability values range between 0.769 and 0.947 and are above the recommended threshold of 0.70 [72]. The Cronbach’s alpha values are higher than 0.70, indicating a suitable internal consistency [72] with two exceptions: cognitive attitude, with a Cronbach’s alpha of 0.617, and perceptions of control with 0.634. There are recommendations stating that if the items’ number is small, the acceptable Cronbach’s alpha value can be above the threshold of 0.5; we keep the latent constructs in the analysis.

Furthermore, Table 3 also shows that the average variance extracted (AVE) for each variable exceeds 0.50, except for cognitive attitudes, whose value is 0.421. However, according to the work of [73], this AVE is still adequate since the composite reliability coefficient associated with this construct is higher than 0.60 (its actual value is 0.769).

Table A2 (Appendix B) shows that the discriminant validity of the measurement holds. The diagonal values in this table are higher in all cases than the corresponding off-diagonal values. Moreover, none of the off-diagonal correlations is greater than the recommended value of 0.8 [74]. Table A3 (Appendix B) presents the combined loadings and cross-loading of all indicator items of the latent construct. The loadings of all reflective items range from a lower bound of 0.716 to an upper bound of 0.947, thus higher than the required threshold 0.7, and all off-diagonal values are lower than the diagonal value for each block of measures items. Cognitive and affective attitudes were derived using formative measurement since each of them has some contribution in explaining the attitude of the respondent regarding fast-food consumption. We, therefore, decide that convergent validity holds.

### 4.2. The Inner Model

Table 4 presents the estimated coefficients of the model along with the corresponding effect sizes. The amount of variance explained (R^2^) for the endogenous construct, behavioral intention is 0.39. We checked for multicollinearity and found that all VIF values are lower than 2.1, and the average block VIF (AVIF) is 1.441, which is below the recommended threshold of 5. In terms of overall model fit, the standardized root means square residual (SRMR) is 0.08, below the acceptable threshold recommended by the authors of [75].

#### 4.2.1. The TPB Dimensions

Cognitive (β = 0.088, *p* = 0.020) and affective (β = 0.041, *p* = 0.171) attitudes show positive association with the intention to consume fast-food products. However, only cognitive attitudes are statistically significant; therefore, H1a is rejected, and H1b is accepted. As for the predictive power of affective and cognitive attitudes, the latter has a higher effect size (0.088) than the former (0.015), showing that the cognitive attitude exerts a stronger influence on the behavioral intention to consume fast food than the affective attitude. Both injunctive (β = 0.218, *p* < 0.001) and descriptive (β = 0.192, *p* < 0.001) norms are predictors of behavioral intention and show a positive influence on fast-food consumption, a result that supports both H2a and H2b. However, injunctive norms have a higher effect size (0.102) than descriptive norms (0.081), showing that the former are stronger predictors than the latter. From the two dimensions of perceived behavioral control, self-efficacy was excluded since it does not have factor loadings above 0.7. Perception of control shows a negative correlation with behavioral intention; however, the value is not statistically significant (β = −0.024, *p* = 0.288). Therefore, H3a was rejected, while we were not able to test H3b due to the fact that self-efficacy had to be removed from the analysis.

#### 4.2.2. The Control Variables

Facilitating factors have a significant positive association with behavioral intention (β = 0.19, *p* < 0.001), while impeding factors are negatively and significantly correlated with intention (β = −0.095, *p* < 0.014). Therefore, both H4a and b are supported. Other two factors that exert an influence on behavioral intention are self-identification as a healthy eater, which has a significant negative relationship with intention to consume fast food (β = −0.112, *p* < 0.005), and sex, which exerts a positive influence on intention toward fast-food consumption (β = 0.074, *p* < 0.042). Though fear of negative evaluation scale (β = −0.032, *p* < 0.228) and age indicate a negative influence on intention, the values are not statistically significant in predicting one’s behavioral intention, so they are not taken into consideration (β = −0.012, *p* < 0.390). We conclude that H5 supported. Table 5 summarizes our findings in terms of accepted and rejected research hypotheses.

#### 4.2.3. Effect Sizes

In terms of effect size, injunctive norms have the highest contribution to the intention to consume fast food (0.102), thus indicating that interventions on injunctive norms may have a meaningful effect since they are strong enough to be considered relevant from a practical point of view [76]. Still relevant, although with smaller effects, are descriptive norms, facilitating factors (0.080, respectively 0.081), cognitive attitude (0.032), self-identification as a healthy eater (0.039), and with a very small effect, impeding factors (0.025). Sex effect is below the minimum threshold of 0.02, thus too weak to be practically relevant, even though the relationship with intention is statistically significant [77].

## 5. Discussion

We explored determinants of the intention to consume fast food among Romanians, using the theory of planned behavior as the main theoretical background. Facilitating and impeding factors, fear of negative evaluation, and self-identification as a healthy eater were used as additional variables. Our model explains 39% of the variations in behavioral intention, less than the 42% identified in the work of [7], or the 67% identified by the authors of [45], and overall less than the average explanatory power of 44.3% found across different studies by the authors of [47]. However, our study explains more than the 25.7% found in the work of [51] and the 34.7% identified by the authors of [78].

We found that affective attitudes are not significantly related to the intention to consume fast food, while cognitive attitudes positively relate to the intention. Our result clarifies the conflicting evidence provided by extant literature. Studies conducted on Korean [1] and Iranian [79] high school students found that out of the three TPB dimensions, attitude was not a significant predictor for behavioral intention, as opposed to another study conducted on an Iranian sample of high school students [51] where attitude was found as the strongest predictor of the intention to consume fast food. All these studies measures attitude as one single construct. Our study takes the path suggested by the authors of [7] and splits the construct into cognitive attitudes and affective attitudes. We confirm the results of [7] and [44] by showing that only cognitive attitudes predict the intention to consume fast food. The authors of [43] suggest a possible explanation of this result in the sense that they found that affective attitudes significantly predict healthy food consumption, but not energy-dense food consumption.

Furthermore, we found that subjective norms have a positive and significant effect on intention, as other previous studies confirmed [1,51,79]. Nevertheless, subjective norms in our case were split into descriptive and injunctive norms, and both were found significant in predicting intention, unlike the results of [7], where only injunctive norms were significant. Our result shows that the respondents’ intention to consume fast food is affected by how their reference group (i.e., family, friends, colleagues, etc.) think and behave regarding fast-food consumption. This aligns with findings from a study on the psychobiology of appetite: “when we eat with others, we have a natural tendency to use their behavior as a guide” [80]. The results of [1] indicate that family, teachers, and friends are the most influential people among young adults, although friends have a more significant influence on respondents’ decision to consume fast food. The authors of [79] show that parents, especially mothers, are also having a strong influence on children’s decisions to consume a certain type of food. Considering that previous research shows that health experts’ opinions on fast food were not significant predictors of the intention to consume [7], our findings are highly important for public policymakers, especially those who are designing nutrition education programs.

As for the influence of perceived behavioral control on the intention toward fast-food consumption, the results indicate that neither of the two constructs that measured this dimension, namely self-efficacy and perceptions of control, predict the intention to consume fast food. These findings are partially in line with those revealed by the original study [7], which shows that respondents’ perception of control over individual fast-food consumption was not statistically significant in predicting intention to consume fast food. On the other side, the same study found that self-efficacy does have a significant negative relation with behavioral intention (β = −0.27, *p*-value < 0.001), resulting in an inhibition to consume energy-dense food products. Considering that self-efficacy was dropped in our research, we cannot either concur or diverge from this finding.

We found that facilitating factors and impeding factors play an important role in predicting the intention to consume fast food. The intention is higher for those who cannot cook, have more spare time, eat alone, or have cravings. On another side, weight and health status concerns, feelings of guilt, and the price paid for buying fast-food products hinder fast-food consumption. Self-identification as a healthy eater also plays a significant role in inhibiting fast-food consumption and a higher predisposition for a healthier meal or a low-fat diet, a finding also supported by the authors of [7,67].

In terms of effect size, the most significant predictor of the extended TPB model regarding fast-food consumption in our Romanian sample is the injunctive norms. This result aligns with [51], which found that subjective norms are the strongest predictor in the extended TPB model, unlike in the basic TPB model where the attitude was the most important predictor is behavioral intention. In our Romanian sample, this result may be explained by the tendency of the Romanian psychosocial and cultural model to pay excessive attention to what others think about them, especially close people [81]. As an additional factor, global society exerts powerful and pervasive influences on dietary and eating habits, many studies showing that social norms are important players in the development and maintenance of obesity around the world [82].

Considering that subjective norms were the predictor with the highest contribution in terms of effect size and therefore the most suitable for practical interventions, a recommended step for behavioral public policymakers, working on reducing fast-food consumption in Romania, should be the design and implementation of nutrition education programs aiming to change societal norms around food choices. Given the obvious influence of the subjective norms, it would be helpful to highlight, through different marketing platforms, the behavior of specific persons with whom people can identify themselves in order to guide the societal change in a suitable direction [83]. In this sense, celebrities or individuals that have an influence on a considerable number of people (e.g., social media influencers, teachers, etc.) and who adopted a healthier lifestyle or avoided fast-food consumption can be involved to spread the awareness of negative fast-food implications on health. In universities and student campuses, especially in diner rooms, it would be effective to show up messages encouraging people to make healthier food choices, using framing effects (e.g., loss aversion, gain seeking, etc.) Some messages could also include statistics about their colleagues’ healthy food choices, nudging them to compare their food behavior with their colleagues’ behavior, and, consequently, to make a change.

According to our findings, young adults between 18 and 24 years old are the group that consumes the most fast-food products, the majority of them have an income under minimum net wage, and they indicated that price and convenience are some of the factors that influence their decision to consume fast-food. A practical incentive to support better decisions regarding food consumption is to encourage these people to choose healthier menus.

In this sense, a decrease in price for healthier food products would have a positive effect on the intention to buy healthy food products. The advantages and benefits of this incentive are also supported by the authors of [84], who debated in a scientific paper the positive or negative effects of different interventions on promoting healthier ready-to-eat meals. Other intervention proposals regarding healthier alternatives for fast-food products encounter difficulties in collaboration with food outlet/chain manager [85]. In this case, the intervention approach should focus on customers or suppliers, or, in the extreme case, the government could apply taxes for unhealthy food products, such as carbonated drinks and saturated fats, and to subsidize food items containing fruits and vegetables in a significant proportion. This might have an impact on individual dietary habits, leading to an improvement in the overall health status.

The research on fast-food consumption in Romania is almost inexistent, and therefore our results cannot be judged against local theoretical backgrounds or previous findings. Although the subject deserves attention in all age groups, the majority of our respondents are students aged between 18 and 35 reached through convenience combined with snowball sampling. A study on a sample representative may deepen the understanding of what drives fast-food consumption in this age group.

Furthermore, a significant number of scientific papers examine fast-food consumption among teenagers, but there is less known on fast-food consumption among older age groups. Further studies need to address the gap, not only in Romania. Concerning this aspect, one neglected issue is the adult consumer’s perception of the definition of fast food [86], since many adults tend to perceive as fast food only the items served by the well-known Western fast-food chains [87]. However, the fast-food items (e.g., burgers, French fries, fried chicken, pizza, etc.) can also be made at home or served by regular restaurants, among other food items. Consequently, a further study should focus on fast-food products that older people eat, but not necessary at a fast-food restaurant.

Future research should relate eating habits and fast-food consumption with objective, clinical data, able to prove not only the relationships between this type of products and a number of noncommunicable diseases but also to measure the magnitude of the relation, allowing for assessing the concrete impact of reducing fast-food consumption on health.

## 6. Conclusions

The patterns of fast-food consumption in Romania are an unexplored niche, and so are their implications for public health and the economy. This study is the first step toward a better understanding of what drives the Romanian population in their choice for dense food consumption. We not only provide an understanding of the Romanians’ perceptions, motivations to consume, and factors that support fast-food consumption, but we also compare findings obtained in a middle-income country with results obtained in developed countries.

## Figures and Tables

**Table 1:** Measurement items by latent constructs as provided by extant literature.

Latent Structure		Observed Variables
Intention to consume [7]		Frequency of fast-food consumption in the next period: INT1, INT2
Attitude [7]	Cognitive	Adjectives that measure the cognitive attitude toward fast-food consumption: ATT1, ATT2, ATT3, ATT4, ATT5
	Affective	Adjectives that measure the affective attitude toward fast-food consumption: ATT6, ATT7, ATT8, ATT9, ATT10, ATT11, ATT12, ATT13, ATT14, ATT15, ATT16, ATT17
Subjective norms [7]	Injunctive Norms	What other people think about fast-food consumption: SN1, SN2
	Descriptive Norms	How other people behave in regards to fast-food consumption: SN3, SN4
Perceived behavioral control [7]	Perceptions of control	Items that capture perceptions of control: PBC1, PBC2.
	Self-efficacy	Items that capture self-efficacy: PBC3, PBC4
Consideration of Future Consequences [7,58]		Items: CFC1–CFC14
Fear of Negative Evaluation [59]		Items that capture how fearful are people about others’ negative evaluation of their fast-food consumption: FNE1–FNE12
Self-identification as a healthy eater [7]		How much a person self-identifies as a healthy eater: SI1, SI2, SI3, SI4
Control beliefs [7]	Facilitating factors	Items that capture factors that facilitate fast-food consumption: BS17–BS20
	Impeding factors	Items that capture factors that inhibit fast-food consumption: BS21–BS24

**Table 2:** Descriptive statistics.

	Participants in Current Study N (%)
**Sex**	
Male	162 (31%)
Female	370 (69%)
**Age**	
Under 18 years old	1 (0.1%)
18–24 years old	442 (83%)
25–34 years old	53 (9%)
35–44 years old	16 (3%)
45–54 years old	18 (3%)
Above 54 years old	2 (0.3%)
**Education**	
Secondary school	1 (0.1%)
High school	171 (32%)
University	323 (60%)
Post-university	35 (6%)
Post high school	2 (0.1%)
**Income**	
Under 1400 lei	282 (53%)
1401–2400 lei	88 (16%)
2401–3400 lei	71 (13%)
3401–4400 lei	37 (6%)
4401–5400 lei	11 (2%)
Above 5400 lei	43 (8%)
**Provenience**	
Rural	174 (33%)
Urban	358 (67%)

**Table 3:** The reliability of measurement.

Variable	Composite Reliability	Cronbach’s Alpha	Average Variance Extracted (AVE)
Intention to consume fast food	0.935	0.861	0.878
Cognitive attitude	0.769	0.617	0.421
Affective attitude	0.947	0.939	0.603
Injunctive norm	0.877	0.720	0.781
Descriptive norm	0.944	0.882	0.895
Perceptions of control	0.845	0.634	0.732
Facilitating factors	0.837	0.740	0.563
Impeding factors	0.839	0.713	0.636
Fear of negative evaluation	0.943	0.931	0.676
Self-Identity as healthy eater	0.935	0.896	0.828

**Table 4:** Path coefficients and effect sizes, with *p*-values in parentheses.

	Path Coefficients/ Significance	Effect Sizes
Cognitive attitude	0.088 * (*p* = 0.020)	0.032
Affective attitude	0.041 (*p* = 0.171)	0.015
Injunctive norms	0.218 *** (*p* < 0.001)	0.102
Descriptive norms	0.192 *** (*p* < 0.001)	0.081
Perceptions of control	−0.024 (*p* = 0.288)	0.006
Facilitating factors	0.19 *** (*p* < 0.001)	0.080
Impeding factors	−0.095 * (*p* = 0.014)	0.025
Self-identification as a healthy eater	−0.112 ** (*p* = 0.005)	0.039
Fear of negative evaluation	−0.032 (*p* = 0.228)	0.002
Sex	0.074 * (*p* = 0.042)	0.008
Age	−0.012 (*p* = 0.390)	0.002

*** *p*-value < 0.001; ** *p*-value < 0.01; * *p*-value < 0.05.

**Table 5:** Summary of hypothesis testing.

Hypothesis		Supported/Rejected
H1a	Affective attitudes are positively related to the intention to consume fast food	Rejected
H1b	Cognitive attitudes are positively related to the intention to consume fast food	Supported
H1c	Cognitive attitudes are stronger predictors of the behavioral intention to consume fast food than affective attitudes	Supported
H2a	Injunctive norms are positively related to the intention to consume fast food	Supported
H2b	Descriptive norms are positively related to the intention to consume fast food	Supported
H2c	Injunctive norms are stronger predictors of behavioral intention than descriptive norms	Rejected
H3a	Perception of control is negatively related to the intention to consume fast food	Rejected
H3b	Self-efficacy is negatively related to the intention to consume fast food	Untestable
H3c	Perception of control is a stronger predictor of the intention to consume fast food compared with self-efficacy	Untestable
H4	Self-identification as a healthy eater is negatively related to the intention to consume fast food	Supported

## Data Availability

Data is available on request.

## Appendix A

**Table A1:** A review of the measurement items.

Dimension as Included in [7]		Item Abbreviation	Item
Intention to consume		INT 1	On a scale of 1 to 7, where 1 means “strongly disagree” and 7 means “strongly agree”, given your lifestyle and/or taste preferences, how much it is likely that you will eat fast food regularly over the next four weeks?
		INT2	On a scale of 1 to 7, where 1 means “definitely false” and 7 means “definitely true”, how much you are likely to eat fast food regularly over the next month?
Attitude	Affective attitude Cognitive attitude	ATT1	To me, eating fast food frequently is… (followed by 5 pairs of adjectives rated on a 7-point scale) Harmful–beneficial
		ATT2	Quick–time-consuming
		ATT3	Convenient–inconvenient
		ATT4	Unpleasant–pleasant
		ATT5	Cheap–expensive
		ATT6	Eating fast food frequently makes me… (followed by 12 pairs of adjectives rated on a 7-point scale): Happy–unhappy
		ATT7	Self-conscious–self-assured
		ATT8	Inadequate–capable
		ATT9	Enticed–disgusted
		ATT10	Guilty–care-free
		ATT11	Lethargic–energetic
		ATT12	Unashamed–ashamed
		ATT13	Disappointed–gratified
		ATT14	Well–unwell
		ATT15	Content–discontent
		ATT16	Worried–calm
		ATT17	Unenthusiastic–enthusiastic
Subjective norms	Injunctive	SN1	Most people who are important to me think that I should eat fast food regularly
		SN2	Those close to me expect me to eat fast food regularly
	Descriptive	SN3	The people in my life whose opinions I value eat fast food regularly
		SN4	Those who are close to me eat fast food regularly
Perceived behavioral control	Perceptions of control	PBC1	I have complete control over the number of times I will eat fast food over the next month.
		PBC2	How often I will eat fast food over the next month is mostly up to me
Control beliefs	Facilitating factors	BS17	I am more likely to eat fast food if I have cravings
		BS18	I am more likely to eat fast food if I have little spare time
		BS19	Eating alone makes it easier for me to choose fast food.
		BS20	I eat fast food as I cannot cook
	Impeding factors	BS21	Concern about my weight prevents me from eating fast food
		BS22	Concern about my health prevents me from eating fast food.
		BS23	I feel guilty if I eat fast food
Fear of Negative Evaluation		FNE1	I worry about what other people will think of me even when I know it does not make any difference
		FNE3	I am frequently afraid of other people noticing my shortcomings
		FNE5	I am afraid others will not approve of me.
		FNE7	When I am talking to someone, I worry about what they may be thinking about me
		FNE8	I am usually worried about what kind of impression I make.
		FNE10	Sometimes I think I am too concerned about what other people think of me
		FNE11	I often worry that I will say or do the wrong things
		FNE12	I am afraid that people will find fault with me
Self-identification as a healthy eater		SI1	I think of myself as a healthy eater
		SI2	I think of myself as someone who is concerned with healthy eating
		SI3	I think of myself as someone who is concerned with the health consequences of what I eat

## Appendix B

**Table A2:** Square roots of AVE.

Construct	Intention	Cognitive Attitude	Affective Attitude	Injunctive Norm	Descriptive Norm	Perceptions of Control	Facilitating Factors	Impeding Factors	Fear of Negative Evaluation	Self-Identify as Healthy Eater
Intention	0.937	0.353	0.338	0.434	0.405	−0.219	0.413	−0.254	0.055	−0.342
Cognitive attitude	0.353	0.649	0.561	0.198	0.192	−0.147	0.441	−0.278	0.134	−0.421
Affective attitude	0.338	0.561	0.776	0.252	0.217	−0.105	0.274	−0.573	0.006	−0.324
Injunctive norm	0.434	0.198	0.252	0.884	0.539	−0.298	0.280	−0.173	0.016	−0.142
Descriptive norm	0.405	0.192	0.217	0.539	0.946	−0.182	0.232	−0.030	0.029	−0.173
Perceptions of control	−0.219	−0.147	−0.105	−0.298	−0.182	0.856	−0.204	0.101	−0.059	0.225
Facilitating factors	0.413	0.441	0.274	0.280	0.232	−0.204	0.750	−0.077	0.198	−0.359
Impeding factors	−0.254	−0.278	−0.573	−0.173	−0.030	0.101	−0.077	0.797	0.150	0.279
Fear of negative evaluation	0.055	0.134	0.006	0.016	0.029	−0.059	0.198	0.150	0.822	−0.101
Self- identity as a healthy eater	−0.342	−0.421	−0.324	−0.142	−0.173	0.225	−0.359	0.279	−0.101	0.910

**Table A3:** Discriminant validity (intercorrelations) of variable constructs.

	Intention	Cognitive Attitude	Affective Attitude	Injunctive Norm	Perceptions of Control	Descriptive Norm	Fear of Negative Evaluation	Facilitating Factors	Impeding Factors	Self-Identity as a Healthy Eater
INT1	0.937	0.017	−0.014	0.044	−0.021	−0.019	−0.001	−0.007	−0.014	0.009
INT2	0.937	−0.017	0.014	−0.044	0.021	0.019	0.001	0.007	0.014	−0.009
ATT3	−0.063	0.862	−0.173	−0.020	−0.050	0.075	0.014	−0.035	−0.102	−0.011
ATT4	0.042	0.804	0.308	−0.026	0.015	−0.064	−0.008	0.053	0.137	−0.069
ATT1	0.260	0.453	0.241	0.250	0.074	−0.031	0.096	0.026	−0.153	−0.124
ATT2	−0.159	0.607	−0.188	−0.122	0.090	0.094	−0.044	0.119	0.132	0.068
ATT5	−0.003	0.375	−0.247	−0.001	−0.151	−0.150	−0.058	−0.257	−0.087	0.214
ATT6	0.049	0.161	0.705	0.024	0.013	−0.099	0.025	0.095	0.160	−0.033
ATT7	−0.024	−0.097	0.819	−0.030	0.001	0.084	0.031	0.061	−0.132	−0.020
ATT8	0.010	−0.024	0.807	0.016	−0.006	0.040	0.015	0.003	0.026	0.052
ATT11	0.012	0.072	0.739	0.202	−0.048	−0.094	−0.020	−0.102	0.023	0.028
ATT12	−0.064	0.036	0.753	−0.007	0.028	−0.027	−0.057	−0.057	−0.207	0.024
ATT13	0.052	−0.150	0.852	−0.008	0.010	−0.028	0.026	0.058	0.027	−0.011
ATT14	−0.017	0.079	0.853	0.009	−0.008	0.007	0.010	−0.012	−0.004	−0.031
ATT15	0.020	0.074	0.870	−0.043	0.029	0.039	−0.002	−0.039	−0.032	−0.024
ATT16	−0.071	−0.082	0.791	−0.118	0.031	0.089	−0.018	0.015	0.008	0.030
ATT17	−0.002	−0.084	0.797	0.000	−0.020	0.025	−0.019	0.035	0.266	−0.048
ATT9	0.105	0.081	0.590	−0.202	−0.033	−0.002	0.059	0.015	0.166	−0.029
ATT10	−0.050	−0.021	0.696	0.143	−0.012	−0.067	−0.047	−0.079	−0.279	0.067
SN1	−0.039	−0.019	0.054	0.884	−0.016	−0.045	−0.019	0.010	−0.053	0.064
SN2	0.039	0.019	−0.054	0.884	0.016	0.045	0.019	−0.010	0.053	−0.064
PBC1	−0.020	0.068	−0.054	0.091	0.856	0.038	−0.036	−0.091	−0.024	0.076
PBC2	0.020	−0.068	0.054	−0.091	0.856	−0.038	0.036	0.091	0.024	−0.076
SN3	−0.014	−0.002	−0.030	0.053	0.025	0.946	−0.002	0.006	0.003	−0.012
SN4	0.014	0.002	0.030	−0.053	−0.025	0.946	0.002	−0.006	−0.003	0.012
FNE1	0.064	0.021	0.034	0.059	0.040	−0.043	0.820	−0.026	0.090	−0.029
FNE3	0.016	0.004	−0.047	−0.058	−0.060	0.035	0.846	0.022	−0.023	−0.006
FNE5	0.102	−0.094	0.004	0.058	−0.108	−0.060	0.794	0.011	0.045	−0.017
FNE7	−0.010	−0.022	0.098	0.003	0.037	−0.047	0.778	0.055	0.031	0.084
FNE8	−0.061	0.053	−0.002	0.047	0.040	−0.019	0.831	−0.069	−0.017	−0.008
FNE10	−0.096	0.038	0.029	0.030	−0.024	−0.029	0.880	0.014	−0.022	0.023
FNE11	−0.012	−0.011	−0.069	−0.072	0.050	0.084	0.794	0.028	−0.077	−0.018
FNE12	0.006	0.004	−0.044	−0.068	0.026	0.078	0.828	−0.033	−0.024	−0.026
BS17	0.046	0.004	0.076	−0.104	0.114	0.003	−0.022	0.736	−0.046	−0.046
BS18	0.007	−0.175	0.051	0.026	−0.056	0.105	0.044	0.802	−0.037	−0.012
BS19	−0.053	0.050	−0.133	0.200	−0.133	−0.174	0.018	0.716	0.004	0.031
BS20	−0.003	0.138	−0.003	−0.118	0.075	0.051	−0.043	0.743	0.082	0.028
BS21	0.023	−0.034	0.235	0.106	0.016	−0.043	−0.019	−0.043	0.809	−0.138
BS22	0.060	−0.035	0.145	−0.092	0.010	−0.046	−0.061	−0.124	0.814	0.123
BS23	−0.089	0.073	−0.400	−0.015	−0.027	0.094	0.085	0.177	0.769	0.014
SI1	−0.109	−0.048	0.049	0.023	0.017	0.003	0.004	0.032	−0.106	0.887
SI2	0.044	0.048	−0.023	0.019	−0.017	−0.014	−0.001	−0.026	0.021	0.935
SI3	0.061	−0.003	−0.025	−0.042	0.000	0.011	−0.003	−0.004	0.082	0.907

## References

[1] Seo H., Lee S.-K., Nam S. (2011). Factors Influencing Fast Food Consumption Behaviors of Middle-School Students in Seoul: An Application of Theory of Planned Behaviors. Nutr. Res. Pract..

[2] Nyachuba D.G. (2010). Foodborne Illness: Is It on the Rise?. Nutr. Rev..

[3] Scutti S. Foodborne Illness May Be on the Rise. Here’s Why. https://www.cnn.com/2018/07/20/health/food-safety-illness-rise-cdc/index.html.

[4] WHO Obesity and Overweight. https://www.who.int/news-room/fact-sheets/detail/obesity-and-overweight.

[5] Mohammadbeigi A., Asgarian A., Moshir E., Heidari H., Afrashteh S., Khazaei S., Ansari H. (2018). Fast Food Consumption and Overweight/Obesity Prevalence in Students and Its Association with General and Abdominal Obesity. J. Prev. Med. Hyg..

[6] Labensky S., Ingram G.G., Labensky S.R. (2000). Webster’s New World Dictionary of Culinary Arts.

[7] Dunn K.I., Mohr P., Wilson C.J., Wittert G.A. (2011). Determinants of Fast-Food Consumption. An Application of the Theory of Planned Behaviour. Appetite.

[8] IBISWorld Industry Market Research, Reports, and Statistics. https://www.ibisworld.com/default.aspx.

[9] Popkin B.M., Adair L.S., Ng S.W. (2012). Global Nutrition Transition and the Pandemic of Obesity in Developing Countries. Nutr. Rev..

[10] Janssen H.G., Davies I.G., Richardson L.D., Stevenson L. (2018). Determinants of Takeaway and Fast Food Consumption: A Narrative Review. Nutr. Res. Rev..

[11] Li L., Sun N., Zhang L., Xu G., Liu J., Hu J., Zhang Z., Lou J., Deng H., Shen Z. (2020). Fast Food Consumption among Young Adolescents Aged 12–15 Years in 54 Low- and Middle-Income Countries. Glob. Health Action.

[12] Choi N.J. (2003). A Report on Nutrient Content and Consumption Patterns of Fast Food.

[13] Pietrangelo A. 13 Effects of Fast Food on the Body. https://www.healthline.com/health/fast-food-effects-on-body.

[14] Romania TV (2012). RomaniaTV.net 1,2 Milioane de Români Mănâncă Zilnic Produse Fast-Food, de o Jumătate de Miliard de Euro Pe an. https://www.romaniatv.net/1-2-milioane-de-romani-mananca-zilnic-produse-fast-food-de-o-jumatate-de-miliard-de-euro-pe-an_36820.html.

[15] Ce Produse Din Categoria “Fast Food” Preferă Cel Mai Mult Românii?. https://www.forbes.ro/ce-produse-din-categoria-fast-food-prefera-cel-mai-mult-romanii-190439.

[16] Romania: Country Health Profile 2019|En|OECD. https://www.oecd.org/publications/romania-country-health-profile-2019-f345b1db-en.htm.

[17] EAPC Country of the Month—Romania. https://www.escardio.org/Sub-specialty-communities/European-Association-of-Preventive-Cardiology-(EAPC)/Advocacy/Prevention-in-your-country/country-of-the-month-romania.

[18] International Diabetes Federation (IDF) (2020). IDF Europe Members—Romania. https://idf.org/our-network/regions-members/europe/members/154-romania.html.

[19] World Health Organization (2016). WHO Diabetes Country Profiles 2016. http://www.who.int/diabetes/country-profiles/en.

[20] Fila S.A., Smith C. (2006). Applying the Theory of Planned Behavior to Healthy Eating Behaviors in Urban Native American Youth. Int. J. Behav. Nutr. Phys. Act..

[21] Azjen I., Fishbein M. (1980). Understanding Attitudes and Predicting Social Behavior.

[22] Voss K.E., Spangenberg E.R., Grohmann B. (2003). Measuring the Hedonic and Utilitarian Dimensions of Consumer Attitude. J. Mark. Res..

[23] Fishbein M., Ajzen I. (2015). Predicting and Changing Behavior: The Reasoned Action Approach.

[24] Cialdini R.B., Kallgren C.A., Reno R.R. (1991). A focus theory of normative conduct: A theoretical refinement and reevaluation of the role of norms in human behavior. Advances in Experimental Social Psychology.

[25] Thaler R.H., Sunstein C.R. (2009). Nudge: Improving Decisions About Health, Wealth, and Happiness.

[26] French J.R.P., Raven B. (1959). The bases of social power. Studies in Social Power.

[27] Ajzen I. (2020). The Theory of Planned Behavior: Frequently Asked Questions. Hum. Behav. Emerg. Technol..

[28] Nystrand B.T., Olsen S.O. (2020). Consumers’ Attitudes and Intentions toward Consuming Functional Foods in Norway. Food Qual. Prefer..

[29] Boston University School of Public Health The Theory of Planned Behavior. https://sphweb.bumc.bu.edu/otlt/mph-modules/sb/behavioralchangetheories/BehavioralChangeTheories3.html.

[30] Kim Y.G. (2014). Ecological Concerns about Genetically Modified (GM) Food Consumption Using the Theory of Planned Behavior (TPB). Procedia Soc. Behav. Sci..

[31] Al-Swidi A., Huque S.M.R., Hafeez M.H., Shariff M.N.M. (2014). The Role of Subjective Norms in Theory of Planned Behavior in the Context of Organic Food Consumption. Br. Food J..

[32] Alam S.S., Sayuti N.M. (2011). Applying the Theory of Planned Behavior (TPB) in Food Purchasing. Int. J. Commer. Manag..

[33] Vanany I., Soon J., Maryani A., Wibawa B. (2019). Determinants of Halal-Food Consumption in Indonesia. J. Islamic Mark..

[34] Han Y., Hansen H. (2012). Determinants of Sustainable Food Consumption: A Meta-Analysis Using a Traditional and a Structura Equation Modelling Approach. Int. J. Psychol. Stud..

[35] Alam S.S., Ahmad M., Ho Y.-H., Omar N.A., Lin C.-Y. (2020). Applying an Extended Theory of Planned Behavior to Sustainable Food Consumption. Sustainability.

[36] Ting H., de Run E.C., Cheah J.-H., Chuah F. (2016). Food Neophobia and Ethnic Food Consumption Intention: An Extension of the Theory of Planned Behaviour. Br. Food J..

[37] Bakti I.G.M.Y., Sumaedi S., Astrini N.J., Rakhmawati T., Yarmen M., Damayanti S. (2020). Applying the Theory of Planned Behavior in Functional Food Purchasing: A Young Consumers Perception. IOP Conf. Ser. Mater. Sci. Eng..

[38] Dunn K.I., Mohr P.B., Wilson C.J., Wittert G.A. (2008). Beliefs about Fast Food in Australia: A Qualitative Analysis. Appetite.

[39] Abraham S., Martinez M., Salas G., Smith J. (2018). College Student’s Perception of Risk Factors Related to Fast Food Consumption and Their Eating Habits. J. Nutr. Hum. Health.

[40] McDermott M.S., Oliver M., Simnadis T., Beck E.J., Coltman T., Iverson D., Caputi P., Sharma R. (2015). The Theory of Planned Behaviour and Dietary Patterns: A Systematic Review and Meta-Analysis. Prev. Med..

[41] French D.P., Sutton S., Hennings S.J., Mitchell J., Wareham N.J., Griffin S., Hardeman W., Kinmonth A.L. (2005). The Importance of Affective Beliefs and Attitudes in the Theory of Planned Behavior: Predicting Intention to Increase Physical Activity1. J. Appl. Soc. Pyschol..

[42] Trendel O., Werle C.O.C. (2016). Distinguishing the Affective and Cognitive Bases of Implicit Attitudes to Improve Prediction of Food Choices. Appetite.

[43] Payne N., Jones F., Harris P.R. (2005). The Impact of Job Strain on the Predictive Validity of the Theory of Planned Behaviour: An Investigation of Exercise and Healthy Eating. Br. J. Health Psychol..

[44] Payne J.W., Bettman J.R., Koehler D.J., Harvey N. (2004). Walking with the Scarecrow: The Information-Processing Approach to Decision Research. Blackwell Handbook of Judgment and Decision Making.

[45] Mirkarimi K., Mansourian M., Kabir M.J., Ozouni-Davaji R.B., Eri M., Hosseini S.G., Qorbani M., Safari O., Rastgari Mehr B., Noroozi M. (2016). Fast Food Consumption Behaviors in High-School Students Based on the Theory of Planned Behavior (TPB). Int. J. Pediatr..

[46] Van Rongen S., Poelman M.P., Thornton L., Abbott G., Lu M., Kamphuis C.B.M., Verkooijen K., de Vet E. (2020). Neighbourhood Fast Food Exposure and Consumption: The Mediating Role of Neighbourhood Social Norms. Int. J. Behav. Nutr. Phys. Act..

[47] McEachan R.R.C., Conner M., Taylor N.J., Lawton R.J. (2011). Prospective Prediction of Health-Related Behaviours with the Theory of Planned Behaviour: A Meta-Analysis. Health Psychol. Rev..

[48] Darley J.M., Latané B. (1968). Bystander Intervention in Emergencies: Diffusion of Responsibility. J. Pers. Soc. Psychol..

[49] Batson C.D., Powell A.A., Weiner I.B. (2003). Altruism and Prosocial Behavior. Handbook of Psychology.

[50] Cho H. (2006). Influences of Norm Proximity and Norm Types on Binge and Non-binge Drinkers: Examining the Under-examined Aspects of Social Norms Interventions on College Campuses. J. Subst. Use.

[51] Sharifirad G., Yarmohammadi P., Azadbakht L., Morowatisharifabad M.A., Hassanzadeh A. (2013). Determinants of Fast Food Consumption among Iranian High School Students Based on Planned Behavior Theory. J. Obes..

[52] Conner M., Norman P., Bell R. (2002). The Theory of Planned Behavior and Healthy Eating. Health Psychol..

[53] Zeinab J., Gholamreza G., Mehdi Y., Mahmood T., Korush J. (2017). Factors Related to Reduction in the Consumption of Fast Food: Application of the Theory-Based Approaches. J. Public Health Res..

[54] Satia J.A., Galanko J.A., Siega-Riz A.M. (2004). Eating at Fast-Food Restaurants Is Associated with Dietary Intake, Demographic, Psychosocial and Behavioural Factors among African Americans in North Carolina. Public Health Nutr..

[55] Carfora V., Caso D., Conner M. (2016). The Role of Self-Identity in Predicting Fruit and Vegetable Intake. Appetite.

[56] Voinea L., Vrânceanu D.M., Filip A., Popescu D.V., Negrea T.M., Dina R. (2019). Research on Food Behavior in Romania from the Perspective of Supporting Healthy Eating Habits. Sustainability.

[57] Naughton S.S., Mathai M.L., Hryciw D.H., McAinch A.J. (2015). Australia’s Nutrition Transition 1961–2009: A Focus on Fats. Br. J. Nutr..

[58] Strathman A., Gleicher F., Boninger D.S., Edwards C.S. (1994). The Consideration of Future Consequences: Weighing Immediate and Distant Outcomes of Behavior. J. Personal. Soc. Psychol..

[59] Leary M.R. (1983). A Brief Version of the Fear of Negative Evaluation Scale. Personal. Soc. Psychol. Bull..

[60] Murphy L., Cadogan E., Dockray S. (2020). The Consideration of Future Consequences: Evidence for Domain Specificity Across Five Life Domains. Pers. Soc. Psychol. Bull..

[61] Psych K.D.B. (2008). Fast-Food Consumption: Application and Extension of the Theory of Planned Behaviour to Incorporate Affective Responses and Implicit Associations. Ph.D. Thesis.

[62] Van Beek J., Antonides G., Handgraaf M.J.J. (2013). Eat Now, Exercise Later: The Relation between Consideration of Immediate and Future Consequences and Healthy Behavior. Personal. Individ. Differ..

[63] Fisher H., Erasmus A.C., Viljoen A.T. (2016). Young Adults’ Consideration of Their Food Choices a Propos Consequences for Their Future Health: Young Adults’ Consideration. Int. J. Consum. Stud..

[64] Gilbert N., Meyer C. (2005). Fear of Negative Evaluation and Eating Attitudes: A Replication and Extension Study. Int. J. Eat. Disord..

[65] Latimer A.E., Ginis K.A.M. (2005). The Importance of Subjective Norms for People Who Care What Others Think of Them. Psychol. Health.

[66] DeBoer L.B., Medina J.L., Davis M.L., Presnell K.E., Powers M.B., Smits J.A.J. (2013). Associations Between Fear of Negative Evaluation and Eating Pathology During Intervention and 12-Month Follow-Up. Cogn. Ther. Res..

[67] Armitage C.J., Conner M. (1999). Distinguishing Perceptions of Control from Self-Efficacy: Predicting Consumption of a Low-Fat Diet Using the Theory of Planned Behavior1. J. Appl. Soc. Psychol..

[68] Cook A.J., Kerr G.N., Moore K. (2002). Attitudes and Intentions towards Purchasing GM Food. J. Econ. Psychol..

[69] Smith J.R., Terry D.J., Manstead A.S.R., Louis W.R., Kotterman D., Wolfs J. (2007). Interaction Effects in the Theory of Planned Behavior: The Interplay of Self-Identity and Past Behavior. J. Appl. Soc. Psychol..

[70] Joreskog K.G., Wold H., Joreskog K.G., Wold H. (1982). The ML and PLS techniques for modeling with latent variables: Historical and comparative aspects. Systems under Indirect Observation: Part I.

[71] Hair J.F. (2010). Multivariate Data Analysis.

[72] Nunnally J.C., Bernstein I.H. (1994). Psychometric Theory.

[73] Fornell C., Larcker D.F. (1981). Evaluating Structural Equation Models with Unobservable Variables and Measurement Error. J. Mark. Res..

[74] Kennedy P. (2008). A Guide to Econometrics.

[75] Hu L., Bentler P.M. (1999). Cutoff Criteria for Fit Indexes in Covariance Structure Analysis: Conventional Criteria versus New Alternatives. Struct. Equ. Modeling A Multidiscip. J..

[76] Cohen J. (1988). Statistical Power Analysis for the Behavioral Sciences.

[77] Kock N. (2014). Advanced Mediating Effects Tests, Multi-Group Analyses, and Measurement Model Assessments in PLS-Based SEM. Int. J. e-Collab..

[78] Didarloo A., Khalili S., Aghapour A.A., Mousavi S.M. (2021). Determining Intention, Fast Food Consumption and Their Related Factors Among University Students by Using A Behavior Change Theory. BMC Public Health.

[79] Ebadi L., Rakhshanderou S., Ghaffari M. (2018). Determinants of Fast Food Consumption among Students of Tehran: Application of Planned Behavior Theory. Int. J. Pediatr..

[80] Higgs S. (2015). Social Norms and Their Influence on Eating Behaviours. Appetite.

[81] David D. (2015). Psihologia Poporului Român: Profilul Psihologic al Românilor Într-o Monografie Cognitiv-Experimentală.

[82] Higgs S., Thomas J. (2016). Social Influences on Eating. Curr. Opin. Behav. Sci..

[83] Ham M., Jeger M., Ivković A.F. (2015). The Role of Subjective Norms in Forming the Intention to Purchase Green Food. Econ. Res. Ekon. Istraživanja.

[84] Hillier-Brown F.C., Summerbell C.D., Moore H.J., Routen A., Lake A.A., Adams J., White M., Araujo-Soares V., Abraham C., Adamson A.J. (2017). The Impact of Interventions to Promote Healthier Ready-to-Eat Meals (to Eat in, to Take Away or to Be Delivered) Sold by Specific Food Outlets Open to the General Public: A Systematic Review. Obes. Rev..

[85] Goffe L., Penn L., Adams J., Araujo-Soares V., Summerbell C.D., Abraham C., White M., Adamson A., Lake A.A. (2018). The Challenges of Interventions to Promote Healthier Food in Independent Takeaways in England: Qualitative Study of Intervention Deliverers’ Views. BMC Public Health.

[86] Musaiger A.O. (2014). Consumption, Health Attitudes and Perception Toward Fast Food Among Arab Consumers in Kuwait: Gender Differences. Glob. J. Health Sci..

[87] Aloia C.R., Gasevic D., Yusuf S., Teo K., Chockalingam A., Patro B.K., Kumar R., Lear S.A. (2013). Differences in Perceptions and Fast Food Eating Behaviours between Indians Living in High- and Low-Income Neighbourhoods of Chandigarh, India. Nutr. J..

